# Digital droplet PCR for precise quantification of human T-lymphotropic virus 1 proviral loads

**DOI:** 10.1186/1742-4690-11-S1-P13

**Published:** 2014-01-07

**Authors:** Giovanna S Brunetto, Raya Massoud, Joan Ohayon, Kaylan Fenton, Irene Cortese, Steven Jacobson

**Affiliations:** 1 Viral Immunology Section, Neuroimmunology Branch, National Institutes of Neurological Disorders and Stroke, Bethesda, MD, USA

## 

Elevated HTLV-1 proviral load (PVL) is thought to be the major risk factor for developing HAM/TSP in HTLV-1 infected subjects, and a high cerebrospinal fluid (CSF) to peripheral blood mononuclear cells (PBMCs) PVL ratio might be diagnostic of the condition. However, the standard method for quantification of HTLV-1 PVL, Real time PCR, has multiple limitations: the inter-assay variability increases at low PVL and low cell numbers in CSF often precludes accurate quantification. Thus, we are evaluating a novel technique, Digital Droplet PCR (ddPCR), as a potentially more reliable tool. For ddPCR, DNA samples are partitioned into thousands of nanoliter -sized droplets, amplified on a thermocycler, queried for fluorescent signal and normalized to a housekeeping gene. Due to the high number of DNA molecules and number of “independent” events (droplets), Poisson algorithms are used to determine absolute copy numbers and are independent of a standard curve. Our results suggest that ddPCR is very accurate: Intraassay variability evaluated by calculating the coefficient of variation of ten replicates of three samples of DNA in three different ranges of PVL (low < 5%, medium 5-10%, and high > 10%) was 13.0%, 7.1% and 9.5%, respectively. Interassay variability, was evaluated by calculating the CV of duplicates of PVL from three independent runs and three independent extractions was 4.5% with a standard deviation of 0.008. Additionally, ddPCR is reliable in quantifying PVL in the CSF where we have confirmed and extended previous observations of increased HTLV-I PVL in CSF of HAM/TSP compared to the periphery.

